# Is Endoscopic Resection a Viable Option for Caecal Leiomyomas Managed Surgically?

**DOI:** 10.7759/cureus.90162

**Published:** 2025-08-15

**Authors:** Maheen Rana, Patricia Pantilie, Robert Hannon

**Affiliations:** 1 General and Colorectal Surgery, Beacon Hospital, Dublin, IRL

**Keywords:** cecal leiomyoma, cecal lesion, colon leiomyoma, endoscopic resection, leiomyoma

## Abstract

Caecal leiomyomas are rare gastrointestinal tumours. They are predominantly asymptomatic and typically incidentally diagnosed during colonoscopies. The treatment of choice is complete resection of the lesion. We present a case of a patient with a two-centimetre caecal leiomyoma who underwent right hemicolectomy. Although there are limited reported cases of caecal leiomyomas undergoing endoscopic resection, our literature review suggests that caecal lesions less than 2 centimetres are generally considered safe for endoscopic resection.

This case underscores the significance of considering less invasive techniques for caecal lesions with a diameter less than 2 centimetres.

## Introduction

Leiomyomas are benign smooth muscle tumours. Gastrointestinal (GI) leiomyomas originate from the layer muscularis mucosae or muscularis propria [1]. They can occur along the entire GI tract, with the oesophagus being the most common site. Colonic leiomyomas are extremely rare, constitute only 3% of all the GI leiomyomas and show a male predominance [2,3]. Among colonic leiomyomas, those on the left side, particularly in the sigmoid colon and rectum, are more frequently reported than those on the right side or in the ascending colon [3].

Leiomyomas are usually discovered incidentally during endoscopic procedures [4]. Endoscopically, they typically appear as sessile intraluminal or intramural tumours with normal overlying mucosa. Mostly asymptomatic, their presentation can vary depending on the size and site of the tumour. The preferred treatment modality is complete resection. The size, location, and presentation of the tumour determine whether endoscopic or surgical resection is more appropriate.

## Case presentation

We present the case of a 54-year-old male patient who underwent colonoscopy due to a strong family history of colorectal cancer. The patient was completely asymptomatic at the time of the procedure.

**Figure 1:**
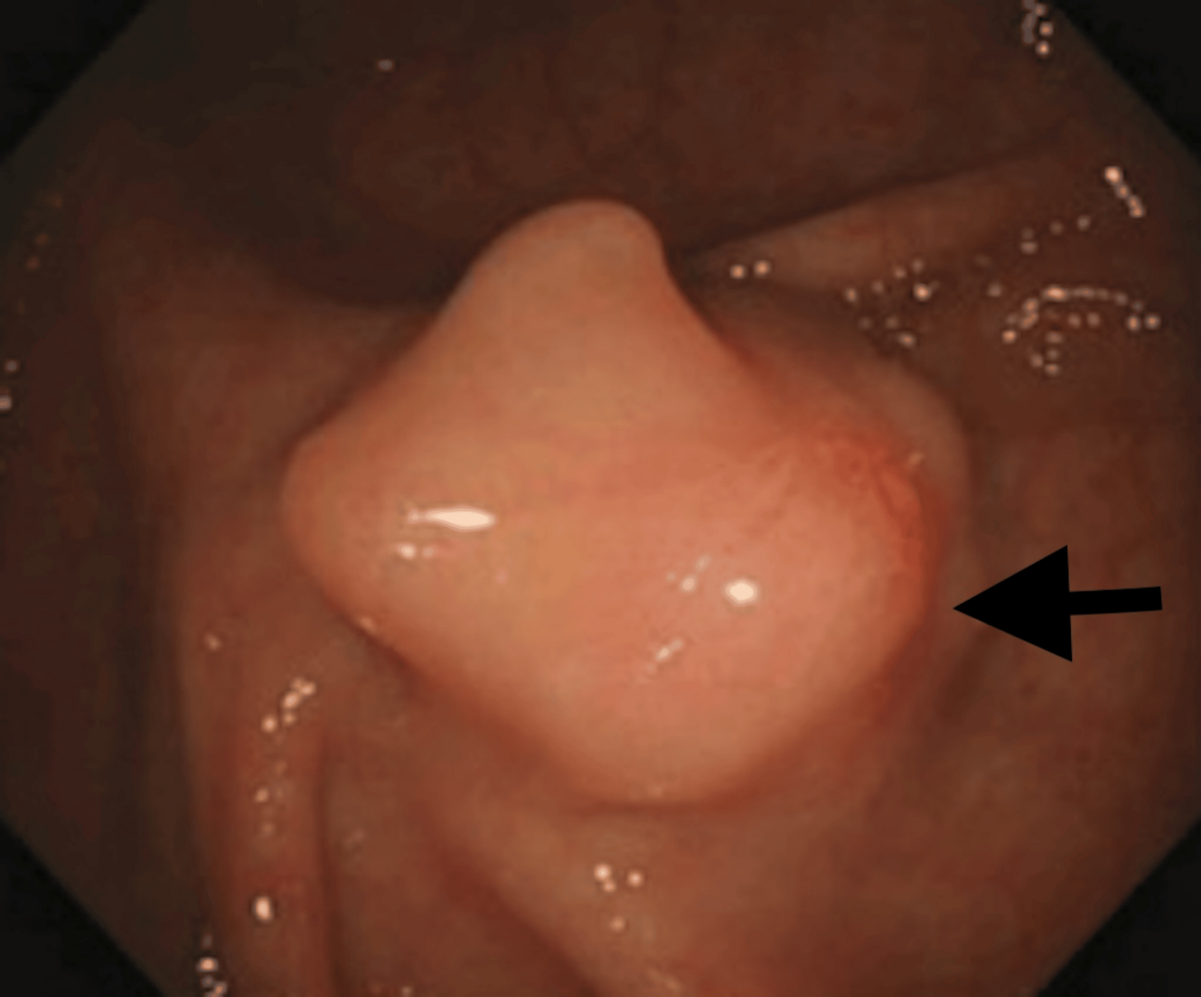
Caecal Polyp

**Figure 2:**
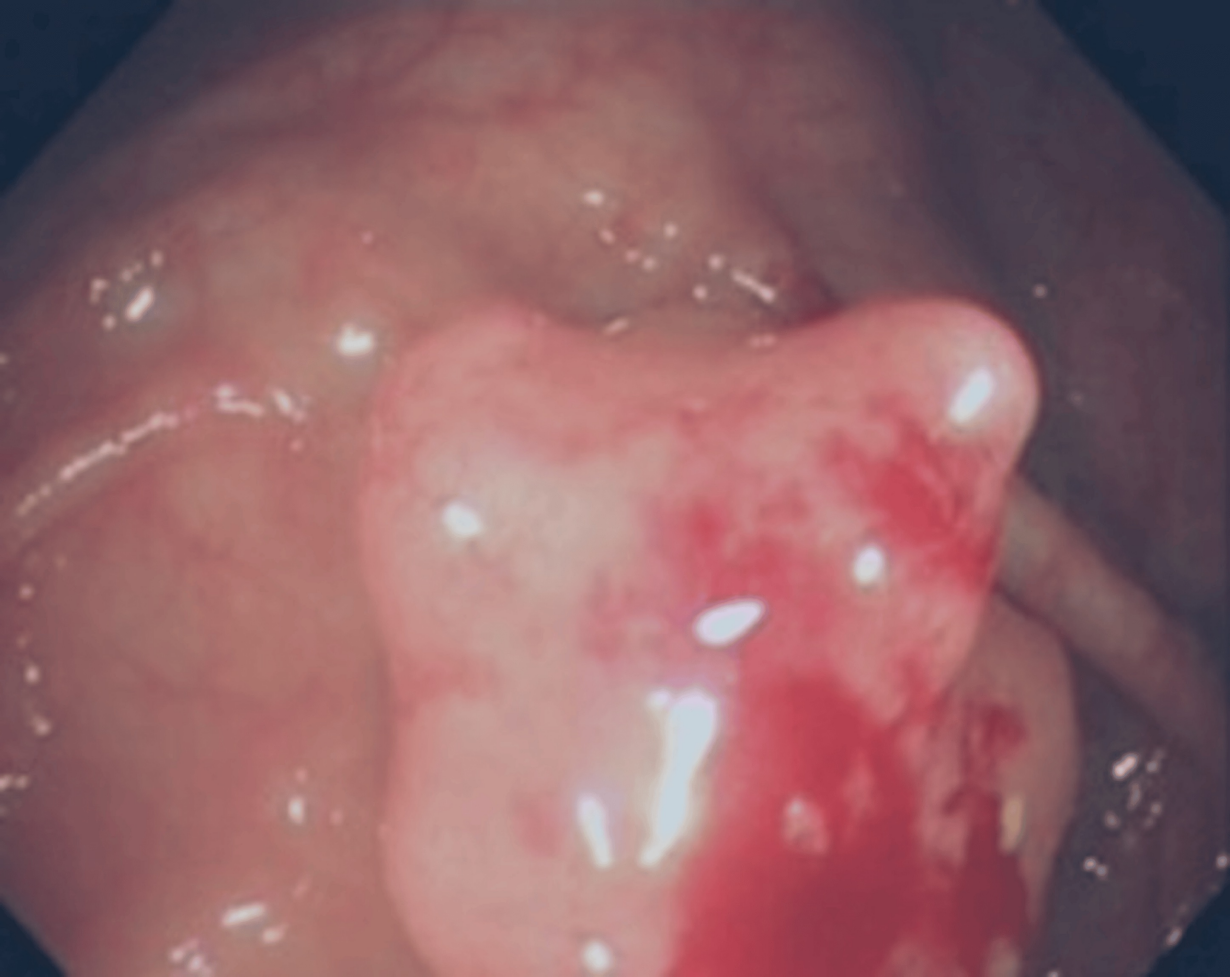
Caecal Polyp From Which Multiple Biopsies Were Taken

The findings of this initial colonoscopy were: a 2cm sessile polyp in the caecum. Multiple biopsies of the lesion were taken and a 3mm sessile polyp in the rectum (completely resected) was seen (Figures 1, 2). 

Regarding the lesion in the caecum, the pathology report revealed normal colonic mucosa with no evidence of adenomatous change or malignancy. The rectal lesion was confirmed to be a hyperplastic polyp. The case was discussed in the Gastrointestinal/Colorectal Oncology multidisciplinary meeting and the decision was made for repeat colonoscopy. During the colonoscopy, multiple biopsies from the caecal lesion were taken (Figure 2).

The pathology report for the second set of biopsies showed fragments of normal mucosa and a fragment of a spindle cell tumour with features suggestive of a gastrointestinal stromal tumour (GIST). The immunohistochemistry report revealed tumour cell expressing Desmin and negative for CD34 and CD117. These features were consistent with a benign leiomyoma. Taking into consideration the size of the lesion and its wide base, the decision for bowel resection was made. The preoperative bloods and scans (CT abdomen, pelvis and thorax) were unremarkable. 

**Figure 3:**
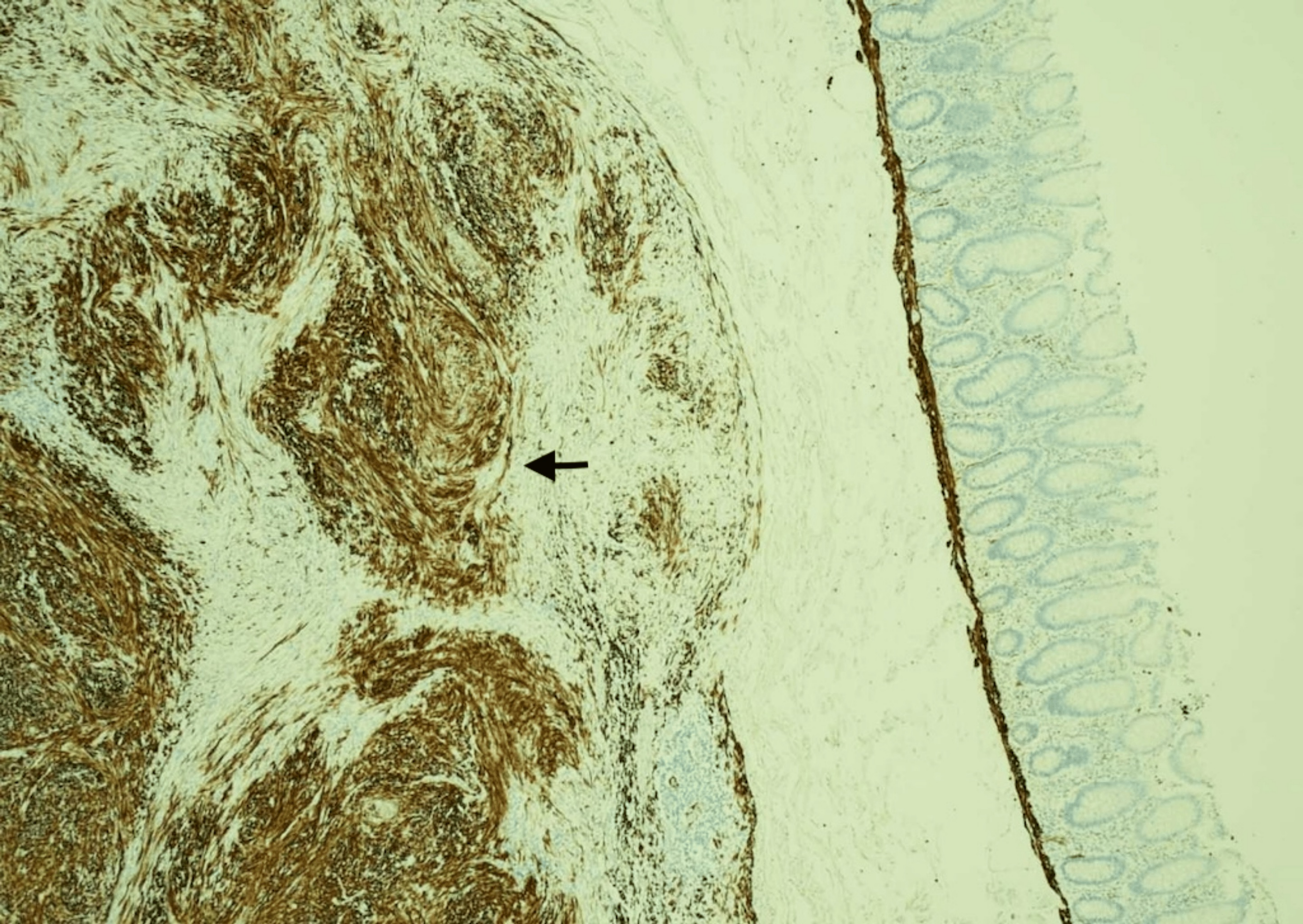
Desmin (x40) Immunohistochemical staining for Desmin (×40). The arrow highlights brown cytoplasmic Desmin positivity within the spindle cells of the leiomyoma, indicating smooth muscle differentiation.

Our patient underwent a laparoscopic right hemicolectomy with ileocolic anastomosis. The postoperative recovery was prolonged since the patient developed postoperative ileus. No other complications followed and the patient was discharged on postoperative day 10. The final pathology report came back as: 20mm well-defined submucosal bland spindled tumour, which is positive with muscle marker (Desmin) (Figure 3), negative with S100 and CD117. CD117 stains some scattered cells admixed. Mitosis and necrosis are not seen. MIB1/Ki-67 immunohistochemistry shows 5%. The findings are most consistent with a leiomyoma. The remainder of the specimen was entirely normal. 

## Discussion

Leiomyomas commonly occur in the female genital tract. Uterine leiomyomas, also known as uterine fibroids, are extremely common benign neoplasms amongst women of reproductive age. In contrast, colonic leiomyomas are rare. These tumours are typically benign and carry a low risk for recurrence after complete removal [5]. 

Endoscopically, leiomyomas present as submucosal, sessile, protruding lesions. Due to smooth, normal-appearing mucosa covering them, it is challenging to characterise these lesions on endoscopic findings alone. CT imaging and endoscopic ultrasound (EUS) can help further evaluate these lesions. An accurate diagnosis usually relies on the combination of endoscopic, radiological (CT or EUS) and histopathological findings [6]. 

Colonic leiomyomas arise from the muscularis mucosae. Histologically, they are composed of well-differentiated smooth muscle cells, lacking mitotic activity or necrosis. Immunohistochemical staining typically shows positive for Desmin and negative for CD34 and CD117, distinguishing them from GISTs [7]. 

The treatment of choice for colonic leiomyomas is complete resection of the lesion. Surgical resection is considered the gold standard treatment, but small lesions <2cm can be successfully treated endoscopically [8]. A retrospective study from Korea reported successful endoscopic resection of leiomyoma, with an average polyp size of 5.82 mm. The study concluded that endoscopic resection is safe for tumours less than 2cm in diameter [8]. Additionally, Lee et al. reported successful endoscopic excision of a 4 cm transverse colon leiomyoma [9]. 

To date, only two published cases of successful endoscopic resection of caecal leiomyomas have been found in our literature review. This may reflect the greater prevalence of colonic leiomyomas in the left colon compared to the right or ascending colon [3]. Badipatla et al. reported a case in 2016 of a 20 mm sessile caecal polyp that was successfully excised endoscopically without post-procedure complications [4]. Another case described the endoscopic removal of a 4 cm semi-pedunculated lesion. Saline injection was used for mucosal lifting before resection, and prophylactic endoclips were applied afterwards. No complications were reported [10].

These lesions can be safely resected using various techniques depending on size, including cold biopsy forceps, polypectomy snares, or endoscopic mucosal resection [8,9,11]. EUS can assist in therapeutic planning by providing detailed information on lesion size, consistency, and depth [12]. Adjunct techniques, such as submucosal lifting via saline injection, can facilitate polyp resection [10]. Additionally, endoclips and endoscopic suturing devices may help manage complications, although their use is dependent on the endoscopist’s experience [8].

## Conclusions

In this case, a right hemicolectomy was performed for a caecal leiomyoma. The primary aim of this report was to review the existing literature to evaluate whether endoscopic resection could be a viable alternative treatment option in selected cases. Although surgical resection remains the standard of care, this case highlights the potential applicability of endoscopic techniques for smaller lesions, particularly those less than 2 cm in size. The feasibility of this approach depends on careful patient selection, the expertise of the endoscopist, and the capabilities of the treating centre, especially in managing potential complications. Further studies are warranted to establish clear treatment guidelines.
